# On-chip crystallization for serial crystallography experiments and on-chip ligand-binding studies

**DOI:** 10.1107/S2052252519007395

**Published:** 2019-06-19

**Authors:** Julia Lieske, Maximilian Cerv, Stefan Kreida, Dana Komadina, Janine Fischer, Miriam Barthelmess, Pontus Fischer, Tim Pakendorf, Oleksandr Yefanov, Valerio Mariani, Thomas Seine, Breyan H. Ross, Eva Crosas, Olga Lorbeer, Anja Burkhardt, Thomas J. Lane, Sebastian Guenther, Julian Bergtholdt, Silvan Schoen, Susanna Törnroth-Horsefield, Henry N. Chapman, Alke Meents

**Affiliations:** a Center for Free-Electron Laser Science, Deutsches Elektronen-Synchrotron DESY, Notkestrasse 85, 22607 Hamburg, Germany; b Photon Science, Deutsches Elektronen-Synchrotron DESY, Notkestrasse 85, 22607 Hamburg, Germany; c Center for Molecular Protein Science, Department of Biochemistry and Structural Biology, Lund University, Kemicentrum, 221 00 Lund, Sweden; d EMBL, Notkestrasse 85, 22607 Hamburg, Germany; e Max Planck Institute of Biochemistry, Am Klopferspitz 18, 82152 Martinsried, Germany; f Bioscience Division and Linac Coherent Light Source, SLAC National Accelerator Laboratory, Menlo Park, CA 94025, USA; g Department of Physics, University of Hamburg, Luruper Chaussee 149, 22761 Hamburg, Germany; h Centre for Ultrafast Imaging, University of Hamburg, Luruper Chaussee 149, 22761 Hamburg, Germany

**Keywords:** serial crystallography, silicon chip, fixed-target crystallography, *in-situ* diffraction, vapor diffusion, ligand binding, ligand soaking, drug discovery, protein structure, X-ray crystallography

## Abstract

Efficient and reliable sample delivery has remained one of the bottlenecks for serial crystallography experiments. Compared with other methods, fixed-target sample delivery offers the advantage of significantly reduced sample consumption and shorter data collection times owing to higher hit rates. Here, a new method of on-chip crystallization is reported which allows the efficient and reproducible growth of large numbers of protein crystals directly on micro-patterned silicon chips for *in-situ* serial crystallography experiments. Crystals are grown by sitting-drop vapor diffusion and previously established crystallization conditions can be directly applied. By reducing the number of crystal-handling steps, the method is particularly well suited for sensitive crystal systems. Excessive mother liquor can be efficiently removed from the crystals by blotting, and no sealing of the fixed-target sample holders is required to prevent the crystals from dehydrating. As a consequence, ‘naked’ crystals are obtained on the chip, resulting in very low background scattering levels and making the crystals highly accessible for external manipulation such as the application of ligand solutions. Serial diffraction experiments carried out at cryogenic temperatures at a synchrotron and at room temperature at an X-ray free-electron laser yielded high-quality X-ray structures of the human membrane protein aquaporin 2 and two new ligand-bound structures of thermolysin and the human kinase DRAK2. The results highlight the applicability of the method for future high-throughput on-chip screening of pharmaceutical compounds.

## Abstract 2

Direct protein crystallization on fixed-target sample holders allows highly efficient serial crystallography experiments and ligand-binding studies.

## 1. Introduction   

Serial crystallography (SX) at X-ray free-electron lasers (XFELs) and, more recently, at the latest low-emittance synchrotron sources has changed the way that data can be collected for X-ray structure determination (Chapman *et al.*, 2011 ▸). In contrast to conventional crystallography, in which structure determination is typically based on diffraction data collection from one or a very few larger crystals that are rotated in the X-ray beam, SX is based on recording still images from several tens to hundreds of thousands of microcrystals (Stellato *et al.*, 2014 ▸; Boutet *et al.*, 2012 ▸; Meents *et al.*, 2017 ▸).

The reliable and efficient delivery of microcrystals into the X-ray beam has remained a bottleneck for SX. Several different sample-delivery approaches have been reported, including liquid jet streaming from gas dynamic virtual nozzles (GDVNs; DePonte *et al.*, 2008 ▸; Weierstall, 2014 ▸), lipidic cubic phase injectors (Weierstall *et al.*, 2014 ▸; Fromme *et al.*, 2015 ▸; Nogly *et al.*, 2016 ▸), different tape drives (Roessler *et al.*, 2013 ▸; Beyerlein *et al.*, 2017 ▸) and solid supports (Zarrine-Afsar *et al.*, 2012 ▸; Hunter *et al.*, 2014 ▸; Roedig *et al.*, 2015 ▸, 2016 ▸; Murray *et al.*, 2015 ▸). Among these, liquid jets have been the most common method for sample delivery to date. Despite being subjected to constant optimization (Oberthuer *et al.*, 2017 ▸), structure determination using liquid jets still requires large amounts of sample, making them inapplicable for more challenging systems, which are often available in small quantities only.

A more promising approach in terms of sample consumption and efficient use of the sample available is the use of solid supports or so-called fixed targets. Here, the microcrystals are located at predefined positions on the substrate and are then raster-scanned through the X-ray beam. This allows efficient high-quality data collection with high hit fractions and low background scattering levels both at XFELs and synchrotrons (Roedig *et al.*, 2015 ▸, 2017 ▸), where the scanning speed is well matched to the measurement rates, which are dictated by the detector frame rates or source repetition rates.

A further challenge for many SX experiments, in particular for ligand-binding studies and also time-resolved experiments, is the need for high-quality microcrystals (Kupitz *et al.*, 2014 ▸). The use of crystals with sizes of only a few micrometres allows the efficient diffusion of a ligand or a substrate into the crystal without substantial deterioration of the crystal quality. Since the diffusion times are in the low-millisecond range (Schmidt & Saldin, 2014 ▸), structural changes involved in ligand binding or enzyme reactions can be studied on at least similar time scales (Stagno *et al.*, 2017 ▸; Kupitz *et al.*, 2017 ▸; Beyerlein *et al.*, 2017 ▸). For time-resolved pump–probe experiments using laser excitation, microcrystals can be excited more homogeneously over the entire crystal volume compared with larger crystals and hence can provide better data (Pande *et al.*, 2016 ▸).

At present, microcrystals for SX are mainly grown by batch crystallization, as this often allows large amounts of well ordered and homogeneously sized samples to be obtained. This approach works well for many small and globular proteins, such as hen egg-white lysozyme. Here, crystal sizes can be well controlled by systematically changing the growth parameters, such as precipitant concentration, pH value and temperature (Falkner *et al.*, 2005 ▸). For other proteins that consist of more than one domain and therefore inherit a certain flexibility, finding the right crystallization conditions is much more challenging. High supersaturation levels often result in protein precipitation. The right crystallization conditions are often found using specially designed sparse-matrix screens in combination with the vapor-diffusion method, which can be automatically set up in 96-well sitting-drop plates using crystallization robots. Compared with batch crystallization, this allows a wider screening of the crystallization space, since both the protein and precipitant concentrations change during equilibration. For such samples, batch crystallization often does not work at all or the conditions cannot be readily concluded from the initially determined sitting-drop crystallization conditions (Chayen, 1998 ▸; Murray *et al.*, 2015 ▸; Wu *et al.*, 2015 ▸). One possible approach to use crystals grown by sitting-drop vapor diffusion for SX experiments is to pool crystals from a large number of crystallization drops. However, this procedure is very time-consuming, induces further crystal-handling steps which can potentially damage the crystals, and is inapplicable for larger crystals as they can no longer be handled as crystal suspensions owing to crystal sedimentation.

A very promising approach for static structure determinations has been developed by Opara and coworkers, in which protein crystals are grown by the vapor-diffusion technique directly on an array of up to 196 silicon nitride membranes ­on a silicon support structure (Opara *et al.*, 2017 ▸). For data collection these 196 compartments need to be sealed with a second silicon nitride membrane, which is placed on top of the silicon support structure, in order to prevent the crystals from drying out. As a consequence, the crystals are not accessible to any chemical reactant after sealing, preventing, for example, micro-diffusion and other time-resolved experiments. A similar approach for on-chip microbatch crystallization between two micro-structured single-crystalline quartz plates has recently been reported by Ren *et al.* (2018 ▸).

We have developed a sitting-drop ‘on-chip crystallization’ approach for fixed-target SX experiments at both synchrotrons and XFELs, in which up to 100 000 crystals can be grown on a single chip. The method is applicable to both batch crystallization and sitting-drop vapor-diffusion experiments. In contrast to other approaches, in which crystals are typically surrounded by large amounts of mother liquor during data collection, our approach allows the mother liquor to be very efficiently removed through micro-pores in the chip substrate, thus yielding ‘naked crystals’. By additionally avoiding any sealing material, this results in much lower background scattering levels and makes the crystals directly accessible to ligand and substrate solutions for ligand-binding studies and time-resolved micro-diffusion experiments. The crystallization chips are furthermore compatible with the fast-scanning Roadrunner gonio­meter, making the method ideally suited to time-resolved experiments at XFELs (Tolstikova *et al.*, 2019 ▸).

In this manuscript, we describe the successful application of this on-chip crystallization approach to six different model proteins: lysozyme, proteinase K, thermolysin, thaumatin, a carbon monoxide dehydrogenase and the human membrane protein aquaporin 2. For three of these proteins, diffraction data were collected. Measurements were performed at synchrotron sources and at the XFEL Linac Coherent Light Source (LCLS) in Stanford, USA. The suitability of our on-chip crystallization system for on-chip ligand-binding studies is demonstrated by the structure solution of two protein–ligand complexes that are new to the Protein Data Bank: thermolysin in complex with aspartate and the human kinase DRAK2 in complex with Mg^2+^-ADP.

## 2. Materials and methods   

### 2.1. Roadrunner silicon chips for SX experiments   

**Figure 1:**
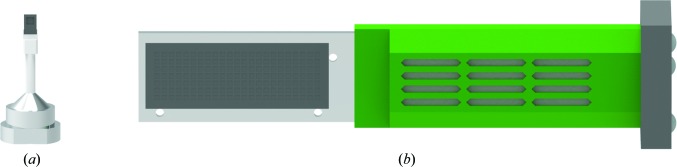
Chip designs for the on-chip crystallization of proteins. (*a*) Roadrunner I chips have overall dimensions of 4.5 × 2.5 mm and up to 10 000 micro-pores, and are equipped with a standard magnetic base, making them compatible with data collection on most MX synchrotron beamlines. (*b*) Roadrunner II chips are much larger, with overall dimensions of 32 × 12 mm, and provide up to 300 000 micro-pores for crystals. They are glued onto an aluminium support frame (shown in light gray), which is mounted on a standard magnetic mount (shown in black). Room-temperature data collection with Roadrunner II chips requires a special scanning stage with a long travel range of more than 32 mm, such as the Roadrunner II goniometer (Tolstikova *et al.*, 2019 ▸). The so-called ‘humidor’ (shown in green) can be slid over the chip to protect the crystals from drying out and from light exposure.

For our experiments two different chip designs were used, which are shown in Fig. 1 ▸. Both chip designs are based on the principle described by Roedig *et al.* (2015 ▸, 2016 ▸). The chips are manufactured from single-crystalline silicon and provide a thin membrane area with a thickness of 10–20 µm equipped with a periodic pattern of micro-pores holding the crystals. The smaller Roadrunner I chips with a membrane area of 1.5 × 1.5 mm^2^ are well suited for cryogenic and room-temperature data collection at synchrotrons and XFELs. They are especially applicable if only limited amounts of sample are available and the crystals typically do not grow too large, as 2–3 µl of crystallization solution is sufficient to cover the entire chip. Owing to their compact design they are further ideally suited for ligand-binding studies and time-resolved experiments at synchrotrons, as the chips are attached to standard magnetic mounts, which are compatible with most MX beamlines. The larger Roadrunner II chips, which are usually loaded with ∼100 µl crystallization solution, are ideally suited if large amounts of sample are available. Roadrunner II chips have already been successfully employed for fast SX data acquisition using the Roadrunner II setup at XFELs such as the MFX and CXI beamlines at LCLS.

### 2.2. On-chip crystallization and data collection using compact Roadrunner I chips   

**Figure 2:**
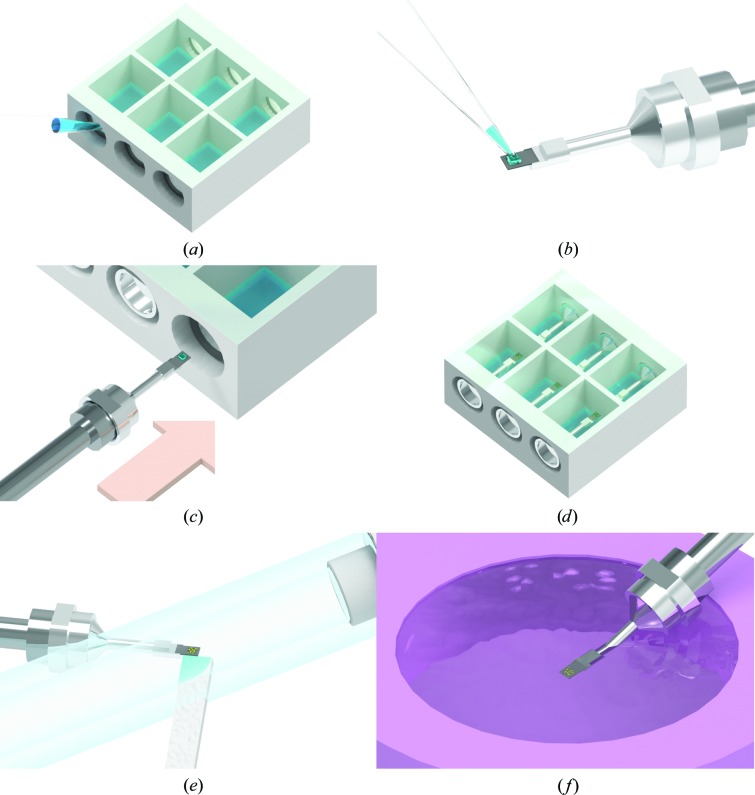
Procedure for on-chip crystallization on Roadrunner I chips. (*a*) For a crystallization experiment, the top of the chamber is sealed with transparent tape before the chamber wells are filled with ≥500 µl reservoir solution through the side opening. (*b*) The protein solution is mixed with precipitant solution and about 2–3 µl of this crystallization mixture is applied to the chip surface. (*c*) The chips are inserted into the crystallization chamber from the side and are held in place by a magnetic ring attached to the lateral opening of the chamber that interacts with the magnetic base of the chip. (*d*) Crystal-growth kinetics are monitored by light microscopy using top light illumination. (*e*) When crystal growth is complete, the chips are taken out of the chamber using a magnetic wand. The excess growth solution is removed through the membrane pores of the chip using a soft tissue, as described by Roedig *et al.* (2015 ▸, 2016 ▸). During all handling steps, starting from the removal of the mother liquor and until room-temperature measurements at the beamline are finished, it is essential to keep the crystals hydrated in a stream of humidified air (Sanchez-Weatherby *et al.*, 2009 ▸; Roedig *et al.*, 2016 ▸). The optimal level of applied humidity has to be tightly controlled and investigated beforehand for each protein individually, as this depends on the buffer composition (Wheeler *et al.*, 2012 ▸). For protein buffers that only contain low concentrations of salt and/or PEG, 98–100% humidity is usually suitable. (*f*) As an alternative to room-temperature measurements, crystals on a chip can be flash-cooled by plunging the chip into liquid nitrogen prior to cryogenic data collection (Roedig *et al.*, 2015 ▸).

Protein crystallization by vapor diffusion requires a closed environment for equilibration between the protein-containing drop and a larger reservoir. As no commercially available system met our needs, we designed chambers for on-chip crystallization on both types of Roadrunner chips. For Roadrunner I chips, a six-well crystallization chamber with dimensions of 60 × 58 × 24 mm (width × length × height) was designed and 3D-printed using polylactic acid as the substrate material. In a typical on-chip sitting-drop crystallization experiment, a 2–3 µl drop of protein–precipitant mixture is pipetted and spread over the silicon chip and subsequently equilibrated against a larger amount of precipitant solution (∼500 µl). The six wells of a crystallization chamber are independent; thus, six different conditions or protein samples can be tested simultaneously. The procedure for a crystallization experiment on Roadrunner I chips is illustrated and described in detail in Fig. 2 ▸.

For feasibility studies, the two proteins proteinase K and thermolysin were crystallized on Roadrunner I chips. Proteinase K crystals with dimensions of 20–30 µm were grown by mixing 15 mg ml^−1^ proteinase K (*Tritirachium album*, Carl Roth) in 0.1 *M* CHC buffer pH 7.0, 10 m*M* CaCl_2_ in a 1:1 ratio with precipitant solution (0.1 *M* CHC buffer pH 6.5, 0.6 *M* ammonium sulfate, 10 m*M* CaCl_2_). 3 µl of this mixture was applied onto a Roadrunner I chip and 500 µl precipitant solution was used as the reservoir. Crystals typically appeared within 12–14 h at room temperature. For on-chip crystallization of thermolysin (*Geobacillus stearothermophilus*, Sigma), the protein was dissolved in 50 m*M* NaOH to a final concentration of 20 mg ml^−1^ and was mixed in a 1:1 ratio with precipitant solution (0.1 *M* MES–NaOH pH 6.5, 10 m*M* CaCl_2_, 25% PEG 2000). 3 µl of this mixture was applied onto a Roadrunner I chip and equilibrated against 500 µl precipitant solution to obtain crystals of ∼35 × 140 µm in size after 1–2 days of incubation at room temperature. Crystal growth was monitored using an Olympus SZX16 microscope, as well as an associated camera and the Olympus *cellSens Standard* software for crystal size measurements.

Data collection from thermolysin crystals grown on Roadrunner I chips was carried out at cryogenic temperatures on beamline P11 at the PETRA III storage ring at DESY (Burkhardt *et al.*, 2016 ▸). After the removal of excess mother liquor, the chips were flash-cooled in liquid nitrogen as illustrated in Figs. 2 ▸(*e*) and 2 ▸(*f*). After cooling, the chips were kept at cryogenic temperature and mounted manually on the goniometer for data collection. For room-temperature measurements of on-chip-grown proteinase K, the mother liquor was removed directly on the beamline and the crystals were kept hydrated by placing the chip in a stream of humidified air as described by Roedig *et al.* (2016 ▸). In both cases, single crystals were manually centered and diffraction data consisting of 30–60° rotation wedges for around 20–30 crystals per chip were measured at 12 keV using a 20 × 20 µm focused beam. Diffraction data were individually processed with *XDS* (Kabsch, 2010 ▸) and merged using the unit-cell cluster analysis in *BLEND* (Foadi *et al.*, 2013 ▸), which included the automatic removal of data affected by radiation damage. Structures were solved by molecular replacement with *Phaser* (McCoy *et al.*, 2007 ▸) using PDB entries 4b5l (J. Jakoncic, V. Stojanoff & V. Honkimaki, unpublished work) and 2tlx (English *et al.*, 1999 ▸) as template structures for proteinase K and thermolysin, respectively. Manual model building and structure refinement were performed in *Coot* (Emsley *et al.*, 2010 ▸) and *PHENIX* (Adams *et al.*, 2010 ▸). Figures of structural models and electron-density maps were prepared with *PyMOL* (DeLano, 2002 ▸).

### 2.3. On-chip crystallization and data collection on Roadrunner II chips   

**Figure 3:**
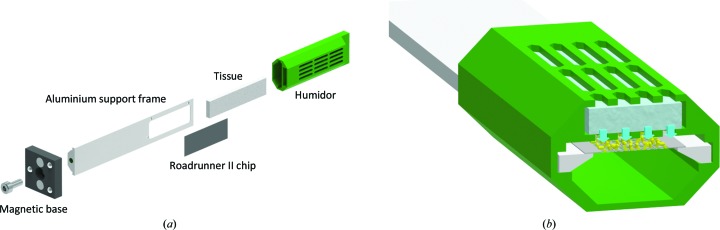
Roadrunner II chip design. (*a*) For assembly, a magnetic base (Thorlabs) is screwed to an aluminium support frame and a Roadrunner II chip is glued on top of the frame cavity. Both assembly steps are assisted by special tools to guarantee perpendicular installation of the base with respect to the support frame and a defined and reproducible position of the chip. The humidor (green) is equipped with a dry piece of tissue and can be slid to the base of the aluminium support frame. (*b*) The humidor was designed to keep naked crystals on the chip in a humid environment. For the short transport between the crystal-preparation laboratory and the beamline, the tissue in the humidor is soaked with water or mother liquor through the gaps on top and the humidor is slid over the chip.

Prior to on-chip crystallization experiments, Roadrunner II chips were glued onto an aluminium support frame to facilitate chip handling. The chip carrying the crystals can be protected using a so-called ‘humidor’. This is a small movable cover that provides a humid environment for the crystals in order to prevent them from drying out during transport. The assembly of a Roadrunner II chip device and the working principle of the humidor are illustrated in Fig. 3 ▸.

**Figure 4:**
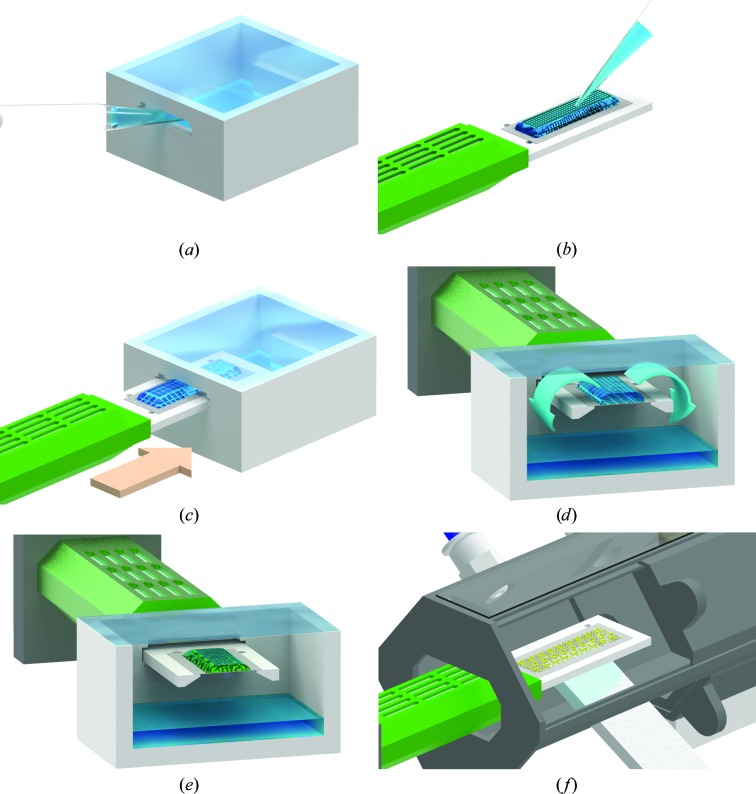
Procedure for on-chip crystallization using Roadrunner II chips. (*a*) The top opening of the crystallization chamber is sealed with transparent sticky tape and the chamber is subsequently filled with 5–10 ml reservoir solution through the side opening. (*b*) 100 µl of the protein–precipitant mixture is applied to the chip and carefully spread over the whole chip area using a pipette tip or a rectangular cover slide. (*c*) The chip holder is then inserted through the side opening of the chamber and the remaining cleft between the chamber and chip holder is sealed with Parafilm to minimize evaporation. (*d*) In the sealed chamber, the vapor-diffusion process leads to a decrease in the drop volume. (*e*) Crystal growth on the chip can be monitored by light microscopy using top light illumination. (*f*) After crystal growth has finished, the chip is removed from the crystallization chamber. Under a stream of humidified air, as shown here in a so-called ‘chip-loading station’, the mother liquor is carefully and thoroughly removed through the pores of the chip with a soft tissue. This handling step can be observed through the transparent window on top of the loading station. Afterwards, the humidor is slid over the chip and the naked crystals can be safely transported to the beamline for data collection.

Following the same design as for Roadrunner I chips, we designed a crystallization chamber for Roadrunner II chips. The chamber has dimensions of 40 × 46 × 21 mm (width × length × height) and can accommodate one chip, which is inserted through a lateral slit at one side of the chamber. The chamber can be filled with up to 10 ml reservoir solution. The procedure for on-chip crystallization on Roadrunner II chips is illustrated in Fig. 4 ▸.

Human aquaporin 2 (hAQP2) was heterologously expressed in *Pichia pastoris* and purified via affinity and size-exclusion chromatography as described previously (Frick *et al.*, 2014 ▸). Purified protein solution in 20 m*M* Tris pH 8.0, 0.3 *M* NaCl, 0.2% octyl glucose neopentyl glycol (OGNPG) was concentrated to 9 mg ml^−1^. The reservoir solution consisted of 0.1 *M* Tris pH 8.5, 0.1 *M* NaCl, 0.1 *M* MgCl_2_, 22–25% PEG 400. For on-chip crystallization, the reservoir solution was diluted in a 4:1 ratio with 0.1 *M* CdCl_2_. The resulting solution was then thoroughly mixed in a 1:1 ratio with protein solution, homogenously applied to the chip and equilibrated against a larger volume of 5–7 ml reservoir solution at room temperature. Crystals grew within 15 min to a few hours.

Carbon monoxide dehydrogenase (CODH) from *Oligotropha carboxido­vorans* was isolated and purified as described previously (Dobbek *et al.*, 1999 ▸; Gremer *et al.*, 2000 ▸). 5 ml reservoir solution composed of 0.75 *M* KH_2_PO_4_/KOH pH 7.5, 0.75 *M* NaH_2_PO_4_/NaOH pH 7.5, 94 m*M* HEPES/NaOH pH 7.5, 3% MPD was used in the experiment. On-chip crystallization was set up by mixing protein solution (19.3 mg ml^−1^ in 50 m*M* HEPES pH 7.0) with reservoir solution containing seeds in a 1:1 ratio. Seeds were obtained by crushing two 250 µm crystals that had previously been grown by conventional hanging-drop crystallization using the same conditions in 50 µl reservoir solution. Crystals grew on the chip at 4°C after approximately 2.5 days.

All other proteins crystallized on Roadrunner II chips were purchased from commercial vendors. Their crystallization conditions are summarized in Table 1 ▸.

Room-temperature data were collected from hAQP2 crystals grown on Roadrunner II chips at the MFX experimental station at LCLS using the Roadrunner II fast-scanning goniometer (Tolstikova *et al.*, 2019 ▸) in combination with the CSPAD detector. The crystals were exposed to a constant stream of humidified helium during data collection to prevent crystal dehydration and to reduce background scattering by air. Data were collected at 9.070 keV with an energy of 2 mJ per 60 fs X-ray pulse and a focal spot size of 1 µm^2^.

After manual alignment, the chips were raster-scanned at a rate of 120 images per second, using a step size of 50 µm and a tilt angle of 25° between the chip-surface normal and the incident X-ray beam in order to avoid incomplete data owing to preferred orientation of the crystals on the chip. One scan of an entire chip took about 15–20 min including chip alignment. We used *OnDa* (Mariani *et al.*, 2016 ▸) to monitor the data collection, *Cheetah* (Barty *et al.*, 2014 ▸) for hit finding and data reduction, *Merge*3*D* to check the orientation bias (Yefanov *et al.*, 2014 ▸) and *CrystFEL* (White *et al.*, 2012 ▸, 2016 ▸) for indexing and merging. The hAQP2 structure PDB entry 4nef (Frick *et al.*, 2014 ▸) served as a model template for structure solution with *Phaser*. The structural model was built and refined in *Coot* and *PHENIX*, respectively (Emsley *et al.*, 2010 ▸; Adams *et al.*, 2010 ▸).

### 2.4. On-chip ligand-binding studies   

**Figure 5:**
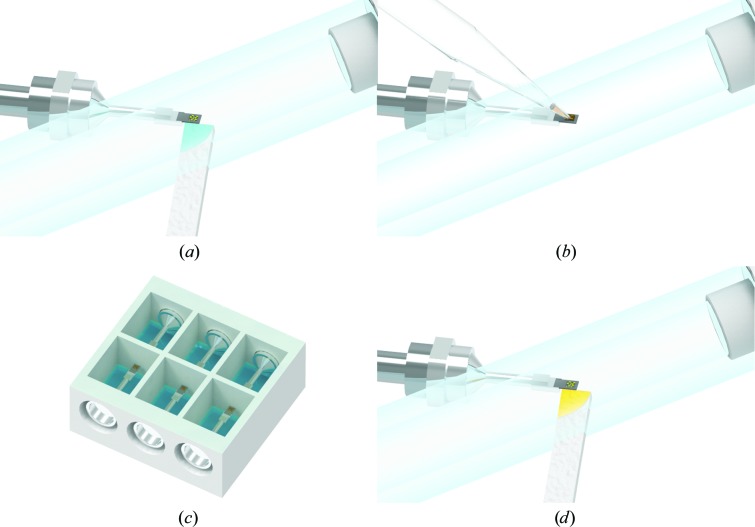
Procedure for on-chip ligand-soaking experiments. (*a*) Crystals are either grown on Roadrunner I chips or 1–3 µl of batch-grown crystals are applied to the chip. Under a stream of humidified air, the mother liquor is removed from the chip through the pores. (*b*) 2–3 µl of ligand-soaking solution is applied to the crystals. (*c*) One well of the crystallization chamber is filled with the appropriate reservoir solution. The chip is installed in the crystallization chamber and incubated for the desired time period. (*d*) The soaking solution is removed through the pores of the chip and crystals are either measured at room temperature or the chip is cooled in liquid nitrogen for cryogenic data collection.

Our crystallization chambers constitute a valuable tool for ligand-soaking experiments, as they provide a stable environment even for extended incubation times of several days to weeks. The procedure for a ligand-soaking experiment on a Roadrunner I chip is illustrated in Fig. 5 ▸. Our setup is not limited to on-chip-grown crystals, but can also be used for protein crystals grown in batch.

We studied the complexes of on-chip crystallized thermolysin with aspartate and in-batch crystallized human serine/threonine kinase 17B (also known as DRAK2) in complex with its natural ligand ATP via on-chip ligand soaking.

Human DRAK2 was expressed and purified in-house following the protocols described on the web pages of the Structural Genomics Consortium (http://www.thesgc.org). In brief, N-terminally His-tagged DRAK2 was expressed in *Escherichia coli*. After cell lysis by ultrasound sonification and centrifugation, the DRAK2 protein in the supernatant was purified via nickel-affinity chromatography and subsequent gel filtration using a Superdex S200 HiLoad column. Fractions containing the DRAK2 protein were pooled and concentrated to ∼20 mg ml^−1^. The final buffer (25 m*M* HEPES pH 7.5, 500 m*M* NaCl, 0.5 m*M* TCEP) was compatible with flash-cooling of the protein and storage at −80°C. Batch crystallization of DRAK2 was performed by mixing 20 mg ml^−1^ thawed protein in a 1:1.5 ratio with 0.2 *M* ammonium acetate, 20% PEG 3350, 50 m*M* sodium/potassium tartrate, 0.8 m*M* quercetin. Crystals of 30–40 µm were ready for experiments after one day of incubation at 20°C.

We soaked thermolysin and DRAK2 crystals for minutes to hours on Roadrunner I chips. We used 200 m*M* sodium aspartate (in 0.1 *M* MES–NaOH pH 6.5, 10 m*M* CaCl_2_, 25% PEG 2000, 25% ethylene glycol) and 50 m*M* ATP (in 0.2 *M* ammonium acetate, 20% PEG 3350, 50 m*M* sodium/potassium tartrate, 25% ethylene glycol) for thermolysin and DRAK2, respectively.

Diffraction data collection at cryogenic temperatures, data processing and structure refinement was performed as described for the Roadrunner I chips (see above). Structures were solved by molecular replacement with *Phaser* (McCoy *et al.*, 2007 ▸) using PDB entries 2tlx (English *et al.*, 1999 ▸) and 3lm5 (Structural Genomics Consortium, unpublished work) as the template structures for thermolysin and DRAK2, respectively.

## 3. Results   

### 3.1. Roadrunner I chips   

**Figure 6:**
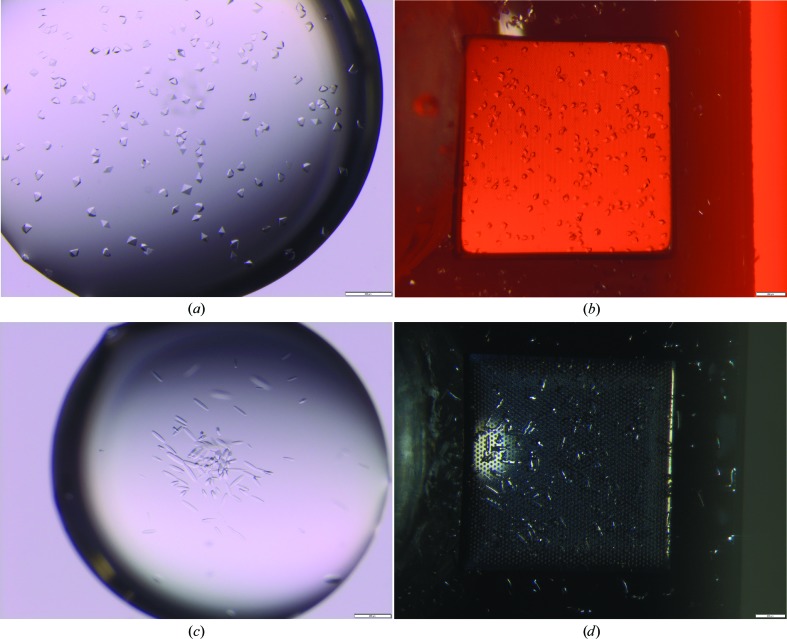
Exemplary results of crystallization experiments for proteinase K (*a*, *b*) and thermolysin (*c*, *d*) using the same crystallization conditions for conventional hanging-drop crystallization (*a*, *c*) and on-chip crystallization on Roadrunner I chips (*b*, *d*). The proteinase K crystals are around 30 µm in size and appeared overnight. The thermolysin crystals needed 1–2 days to reach a maximal size of ∼35 × 150 µm. The scale bars at the bottom right corners each have a length of 200 µm. The different background colors in the pictures showing on-chip crystallization stem from the different polylactic acid colors used for 3D-printing of the crystallization chambers.

In a conventional hanging-drop vapor-diffusion experiment, proteinase K crystals of ∼30 µm appeared overnight at room temperature when 6 µl of a 1:1 mixture of 15 mg ml^−1^ protein solution and precipitant solution consisting of 0.6 *M* ammonium sulfate was equilibrated against 500 µl reservoir solution. Using the same crystallization conditions (3 µl of a 1:1 mixture containing 15 mg ml^−1^ proteinase K equilibrated against 500 µl reservoir solution), we crystallized proteinase K on Roadrunner I chips, resulting in 25–40 µm crystals overnight [Fig. 6 ▸(*b*)]. The crystals were evenly distributed over the whole chip area and remained in place when the mother liquor was removed. We obtained the same result for the crystallization of thermolysin. Using identical crystallization conditions for both conventional hanging-drop experiments and crystallization on Roadrunner I chips, thermolysin crystals were observed to grow reproducibly to a maximal size of around 35 × 150 µm.

Diffraction data from proteinase K and thermolysin crystals were collected on beamline P11 of the PETRA III storage ring (Burkhardt *et al.*, 2016 ▸) using the abovementioned multi-crystal approach at room temperature and cryogenic temperature, respectively (Table 2 ▸).

**Figure 7:**
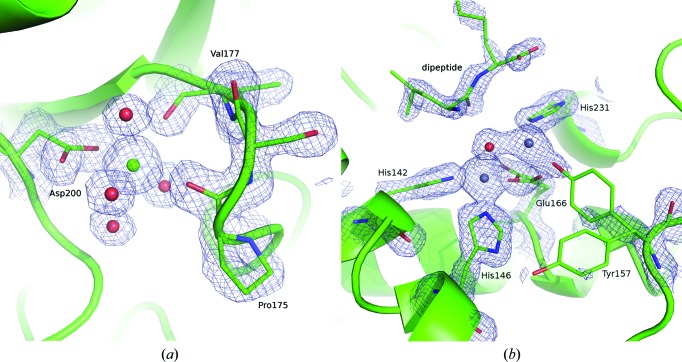
Exemplary 2*mF*_o_ − *DF*_c_ electron densities at 1.5σ for the structures of proteinase K (*a*) and thermolysin (*b*) derived from crystals grown on Roadrunner I chips. The Ca^2+^-binding site of proteinase K and the active site of thermolysin with two bound Zn^2+^ ions (gray spheres) are shown. Residues that are involved in ion binding and/or are functionally important are represented as sticks and labeled. For a more detailed discussion on the thermolysin structure, please see the supporting information.

We solved the room-temperature proteinase K structure at 1.74 Å resolution [Fig. 7 ▸(*a*)] using diffraction data from three 30 µm crystals merged according to *BLEND* cluster analysis and using PDB entry 4b5l as the template for molecular replacement. Comparison with either PDB entry 5avj (Yazawa *et al.*, 2016 ▸), a cryogenic temperature structure of proteinase K at 1.45 Å resolution, or PDB entry 1ic6 (Betzel *et al.*, 2001 ▸), a 0.98 Å resolution room-temperature structure of proteinase K, reveals that our structure is identical to the available structures in the PDB (main-chain r.m.s.d.s of 0.210 and 0.230 Å, respectively), only showing the conformational flexibility of side chains that is typical of room-temperature structures.

For the apo form of thermolysin measured at cryogenic temperature, structure solution and refinement was performed with data merged from three individual crystals using the native thermolysin structure with PDB code 2tlx (English *et al.*, 1999 ▸) as a template for molecular replacement. Our 1.73 Å resolution thermolysin structure superposes well with the reference structure 2tlx, with an r.m.s.d. of 0.273 Å for all main-chain atoms. A more detailed discussion of our thermolysin structure can be found in the supporting information.

### 3.2. Roadrunner II chips   

**Figure 8:**
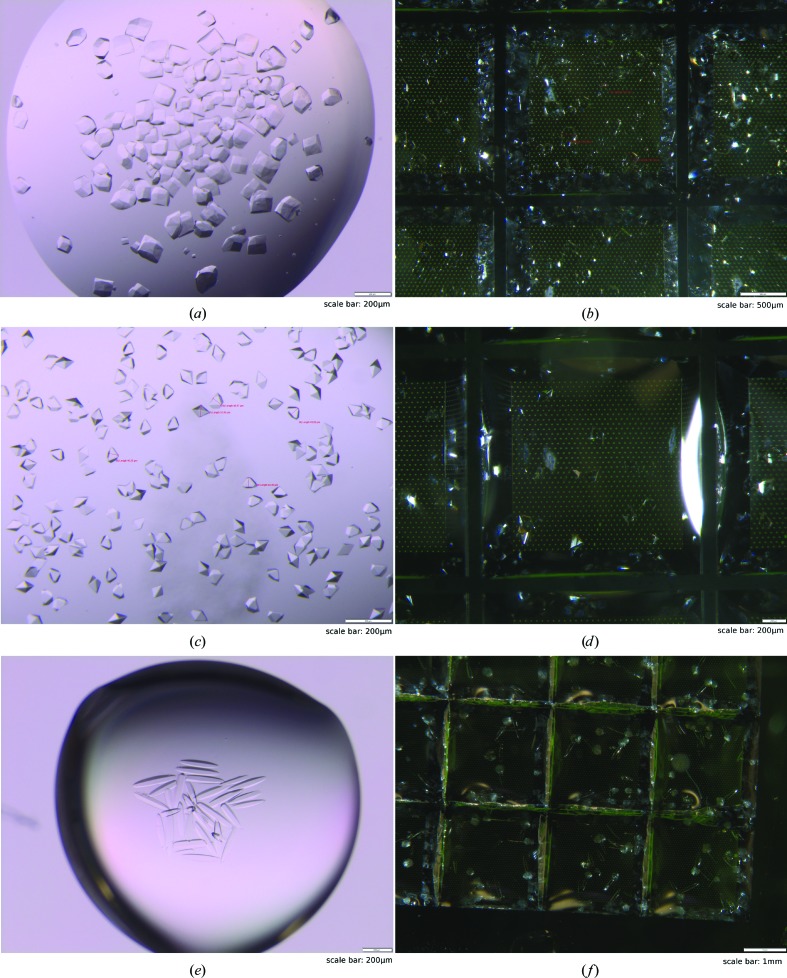
Exemplary images of crystals grown in hanging drops (*a*, *c*, *e*) and on Roadrunner II chips (*b*, *d*, *e*) using the same crystallization conditions. The crystallization conditions and crystal sizes for lysozyme (*a*, *b*), proteinase K (*c*, *d*) and thermolysin (*e*, *f*) are summarized in Table 1 ▸.

We crystallized six different proteins on Roadrunner II chips, including globular proteins such as proteinase K, hen egg-white lysozyme, thaumatin, thermolysin and carbon monoxide dehydrogenase (CODH) as well as the human membrane protein aquaporin 2 (hAQP2). Starting from the optimized hanging-drop crystallization conditions, we obtained crystals that already showed a similar size and shape in the first attempt for all samples. The crystallization conditions as well as the resulting crystal sizes are summarized in Table 1 ▸. In general, the crystal growth and appearance showed the same characteristics as in a conventional vapor-diffusion experiment (Fig. 8 ▸). Protein crystals that exhibited a large diversity in shape and size under a specific crystallization condition, such as thaumatin and hAQP2, also appeared with a similar shape and size distribution on the chips. For almost all samples, crystals grew at a similar speed as in a conventional sitting-drop experiment, ranging from 15 min to one day. Only the growth of CODH crystals on the chip took approximately 2.5 days at 4°C, in contrast to overnight growth in hanging drops.

**Figure 9:**
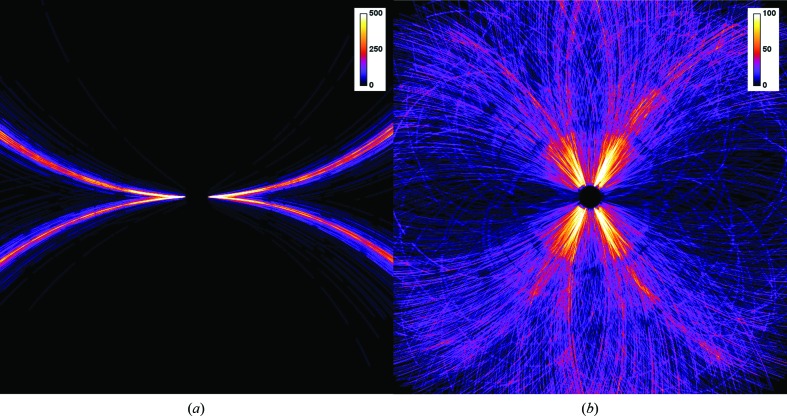
Accumulated Bragg spots of 3000 patterns collected from hAQP2 crystals with the silicon chip oriented perpendicular to the beam (*a*) and at a tilt angle of 25° (*b*). The preferred orientation of the crystals lying on the silicon-chip surface leads to poor coverage of reciprocal space. In order to obtain a more complete data set, the chip has to be tilted during data collection. The color code of the scale bars indicates the number of patterns that contribute to each pixel in merged reciprocal space.

We collected serial diffraction data for on-chip crystallized human aquaporin 2 (hAQP2) at room temperature at LCLS using the fast-scanning Roadrunner II goniometer. From two Roadrunner II chips we obtained 3377 hits from 137 476 collected frames (a 2.5% hit rate), leading to 2723 indexed patterns (an 80.6% indexing rate). The measurement time per chip was about 20 min for the first chip and about 9 min for the second chip, resulting in a total measurement time of around 30 min for the full data set. The rod-shaped hAQP2 crystals showed a strong preference for two distinct orientations rotated along their long fourfold symmetry axis when lying on the flat silicon-chip surface. The corresponding diffraction patterns collected with a beam perpendicular to the chip plane hence show a strong bias and Bragg peaks only cover a very narrow region of reciprocal space (Fig. 9 ▸). In order to obtain a complete data set, we tilted the chip by 25° during SX data collection.

Using these data, the hAQP2 crystal structure was solved to a resolution of 3.7 Å (Table 3 ▸), exhibiting lower overall quality than the cryo-structure reported in the PDB with a resolution of 2.75 Å (PDB entry 4nef; Frick *et al.*, 2014 ▸). In order to exclude any potential negative effect of our on-chip crystallization on the crystal quality, we also measured hAQP2 crystals pooled from sitting-drop plates grown using the same crystallization protocol. These crystals showed the same diffraction properties and quality as the on-chip grown crystals. From previous screening of cryo-cooled hAQP2 crystals we have found a correlation between crystal size and diffraction properties. Therefore, the weaker diffraction properties of the crystals used in this work are most likely to be owing to their significantly smaller size compared with the crystals used for previous structural determination and do not originate from our on-chip crystallization method.

**Figure 10:**
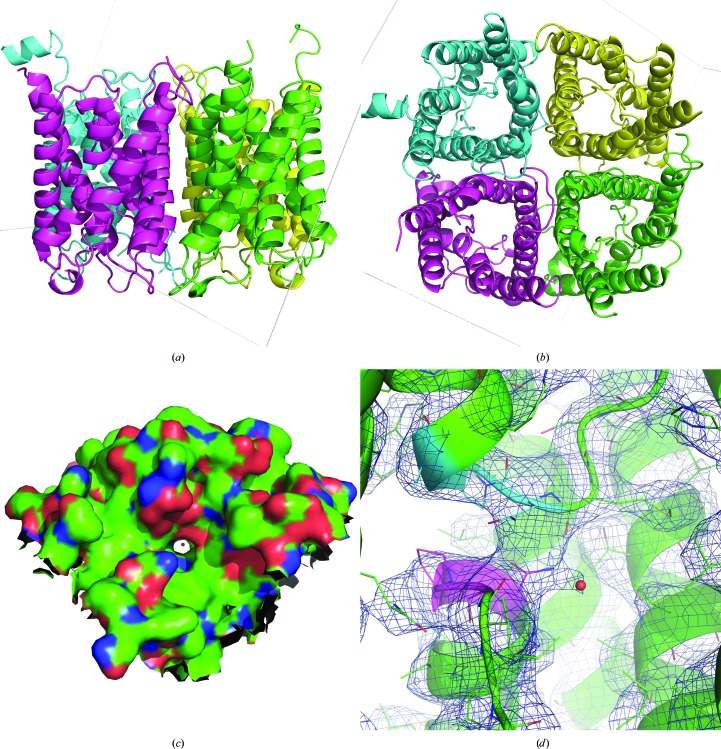
Structure of human aquaporin 2 (hAQP2) obtained by room-temperature SFX from crystals directly grown on Roadrunner II chips. Views are parallel to the membrane plane (*a*) and perpendicular to the membrane plane (*b*) in cartoon representation. hAQP2 forms a homotetramer, with each monomer consisting of six transmembrane helices and two half-helices forming a pseudo-transmembrane helix. Each monomer is shown in a different color for clarity. Gray lines indicate the unit cell axes. (*c*) A water molecule (red sphere) is situated in the middle of the water channel of one monomer. The shown surface represents the solvent-excluded surface area viewed from the extracellular side. (*d*) The view perpendicular to the water channel shows that the water molecule is situated at a hydrogen-bond distance from the side chain of Asn68. This residue is part of the highly conserved dual NPA motif (pink and cyan) in the center of aquaporin channels that plays a central role in channel selectivity. The 2*mF*_o_ − *DF*_c_ electron density is shown at 0.8σ.

Human AQP2 forms a homotetramer in the protein crystal as well as in the biologically active structure (Fig. 10 ▸). Each monomer contains a water channel surrounded by six transmembrane helices and two half-helices forming a pseudo-transmembrane helix. Comparison of our on-chip crystal structure with the reference structure available in the PDB (PDB entry 4nef), which was solved by conventional cryo-crystallography, revealed no major differences in the overall conformation of the tetramer. In particular, the predominantly α-helical and well ordered protein cores of both structures, built by the 4 × 6 transmembrane helices, superimpose well (r.m.s.d. of 0.622 Å for 1936 main-chain atoms), whereas the surface loops naturally show higher flexibility. In general the structures are similar, with an overall r.m.s.d. value of 0.928 Å for all 3596 main-chain atoms. Both of the Cd^2+^-binding sites present in the reference structure PDB entry 4nef were confirmed by our structure. Despite the limited resolution of the data, clear density for a water molecule is observed in the middle of the water-conducting pore of one of the hAQP2 monomers, close to the conserved NPA region. Further details are provided in the supporting information.

### 3.3. On-chip ligand-binding studies   

Our experiments revealed that our on-chip crystallization chambers are very well suited for on-chip ligand-binding experiments. A great advantage is the direct accessibility of the ligand solution to the ‘naked’ crystals on the silicon chips. Following this approach, we were able to solve the crystal structures of on-chip crystallized thermolysin in complex with aspartic acid and in-batch crystallized DRAK2 crystals in complex with ADP, both of which are new structures to the PDB.

**Figure 11:**
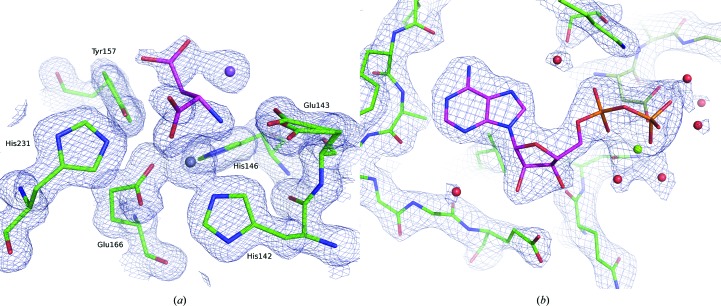
Ligand-binding sites of the structures obtained from on-chip ligand-soaking experiments using our crystallization chambers and Roadrunner I chips. (*a*) An aspartate molecule (pink) in complex with a sodium ion (purple sphere) is bound to the active site of thermolysin. It is involved in the fivefold coordination of the catalytic zinc ion (gray sphere) with both its amino and carboxy moieties. The 2*mF*_o_ − *DF*_c_ electron-density map at 1σ is shown as a blue mesh and catalytically important thermolysin residues (green) are labeled. (*b*) The 2*mF*_o_ − *DF*_c_ electron-density map at 1.5σ clearly indicates the presence of Mg^2+^-ADP (yellow sphere and pink sticks) at the active site of DRAK2.

Apo crystals of thermolysin were grown directly on Roadrunner I chips and were subsequently soaked with 200 m*M* aspartate dissolved in mother liquor, using our on-chip crystallization chambers in both steps. Diffraction data were measured at cryogenic temperature. Structure solution and refinement was performed using a 1.52 Å resolution data set obtained by merging data from five crystals, using the native thermolysin structure (PDB entry 2tlx; English *et al.*, 1999 ▸) as a template for molecular replacement. At the active site we observed two bound aspartate molecules, one of which is involved in the pentacoordination of the catalytic zinc ion [Fig. 11 ▸(*a*)]. The binding mode of this aspartate molecule is similar to that of asparagine bound to thermolysin (PDB entry 4m65; Yin *et al.*, 2014 ▸). Details are provided in the supporting information.

DRAK2 is a serine/threonine kinase that is involved in autoimmune diseases and cancer (Sanjo *et al.*, 1998 ▸; Doherty *et al.*, 2009 ▸; Yang *et al.*, 2012 ▸; Edwards *et al.*, 2015 ▸; Harris & McGargill, 2015 ▸). DRAK2 crystals were grown by batch crystallization and were soaked on a Roadrunner I chip for 1 h with the natural ligand ATP using the on-chip crystallization chamber. Cluster analysis of the collected data with *BLEND* led to a 2.5 Å resolution data set merged from four individual crystals. The crystal structure was solved via molecular replacement using the quercetin-bound structure of DRAK2 (PDB entry 3lm5) as a search model. At the active site, electron density for the nucleotide and one coordinated magnesium ion is clearly visible, although the lack of density for the γ-phosphate indicates that ATP was hydrolyzed to ADP during incubation [Fig. 11 ▸(*b*)]. A more detailed description of the active site and a comparison with structures of the related kinase DAPK1 are provided in the supporting information.

## 4. Conclusion and outlook   

### 4.1. On-chip crystallization   

The recent evolution of high-intensity X-ray techniques such as serial femtosecond crystallography (SFX) at XFELs goes hand in hand with the need for extremely fast sample exchange. Fixed-target sample holders are often advantageous over liquid-jet and LCP systems, as they typically lead to much higher hit fractions, provide lower background levels and work more reliably, hence causing significantly reduced downtimes of the instrument. Probably the most important advantage is that they require orders of magnitude less sample.

Here, we present a new method for *in-situ* protein crystallization directly on silicon chips, which provides a straightforward and highly reliable method for sample delivery for SX experiments at synchrotrons and XFEL facilities. It constitutes an alternative approach to batch crystallization, which, owing to the harsh conditions, is not always possible for all proteins. It also avoids time-consuming crystal pooling from tens to hundreds of individual crystallization drops. Known hanging-drop or sitting-drop crystallization conditions can be directly transferred to the on-chip crystallization method and in most cases do not require any further optimization.

Loading of pre-grown protein crystals onto fixed-target sample holders in an efficient way has still remained challenging, as this involves additional crystal-handling steps and often leads to crystal clustering when the surrounding liquid is removed (Soares *et al.*, 2014 ▸). The on-chip crystallization method is of particular interest for soft and fragile protein crystals. These often suffer from shearing forces during pipetting, which is now avoided since they are measured *in situ* on their crystallization substrate. In general, we even expect a higher crystal quality for the on-chip grown crystals owing to the reduced crystal-handling steps. In addition, the on-chip grown crystals remain in place upon the removal of the mother liquor, showing no clustering. Hence, we achieve a more homogenous distribution of the crystals on the chip for SX measurements, preventing multiple crystals per exposure.

The hit rates of 2.5% as observed for hAQP2 on the Roadrunner II chips were low for a fixed-target experiment and can probably be attributed to the low protein concentration that was used. The number of crystals on the chip could be increased by using a larger amount of crystallization solution, as Roadrunner II chips have a high capacity of at least 120 µl of solution. An alternative approach for increasing the crystal density is to vary the crystallization parameters such as the protein and precipitant concentration. This strategy was not applicable for hAQP2 as it causes unwanted changes in the crystal morphology. However, even though the hit rates were low, a total data collection of 30 min still appears to be reasonable for an SFX structure determination.

### 4.2. On-chip ligand binding   

X-ray crystallography allows the study of the interaction of pharmaceutical target proteins with potential drugs on the atomic level, which often requires the screening of hundreds to thousands of different compounds. To date, most of these ligand-binding experiments have been performed manually using conventional crystallography. For these studies, relatively large crystals are individually harvested from their growth solution, transferred to the solution containing the ligand and subsequently flash-cooled for cryogenic data collection (Schiebel *et al.*, 2016 ▸). These steps have to be repeated for every ligand solution. Consequently, these studies are a very tedious and time-consuming procedure. Even though efforts have been undertaken to automate the crystal-harvesting step (Deller & Rupp, 2014 ▸; Samara *et al.*, 2018 ▸), the whole procedure remains a major bottleneck for screening of entire chemical libraries. Here, we show that fixed-target serial crystallography provides a very promising alternative to these conventional ligand-binding studies. In contrast to current practice, our method avoids most of the crystal-handling steps as the crystals are grown and soaked with the ligand solution directly on their support structure and measured *in situ*.

The Roadrunner crystallization chambers provide a well suited environment for ligand-binding experiments, as we show for the binding of aspartate to thermolysin and for the DRAK2–ADP complex. In difficult cases, where the desired compound is sparsely soluble, our system has the advantage that the mother liquor can be completely removed prior to application of the ligand solution. Hence, the ‘naked’ crystals can be exposed to the highest possible ligand concentrations.

Furthermore, Roadrunner I chips are compatible with data collection at most synchrotron MX beamlines, as the chips are mounted on commonly used standard magnetic bases. In combination with sample-changing robots and automated raster-scanning procedures of the chips, which are increasingly available at synchrotron beamlines such as DESY beamline P11 (Burkhardt *et al.*, 2016 ▸), this should allow much faster and more efficient ligand-binding studies at synchrotron sources. The use of polychromatic ‘pink’ X-ray beams providing much higher X-ray intensities should make data collection even faster. The larger bandwidth of up to 5% for a polychromatic X-ray beam has been shown to further significantly reduce the number of crystals and thus the amount of sample required to obtain a complete data set. In a recent pink-beam diffraction experiment, still diffraction images collected from only 50 microcrystals were sufficient for protein structure determination (Meents *et al.*, 2017 ▸). Hence, the combination of pink-beam SX, on-chip crystallization and on-chip ligand-binding experiments holds huge potential for high-throughput ligand screening of pharmaceutically relevant target proteins.

## 5. Related literature   

The following references are cited in the supporting information for this article: de Diego *et al.* (2010 ▸), Holland *et al.* (1995 ▸), Temmerman *et al.* (2014 ▸) and Thorn & Sheldrick (2011 ▸).

## Supplementary Material

PDB reference: proteinase K crystallized on a silicon chip, 6qf1

PDB reference: thermolysin crystallized on a silicon chip, 6qf2

PDB reference: thermolysin soaked with sodium aspartate on a silicon chip, 6qf3

PDB reference: human serine/threonine kinase 17B in complex with ADP obtained by on-chip soaking, 6qf4

PDB reference: human aquaporin 2 crystallized on a silicon chip, 6qf5

Supplementary information and figures. DOI: 10.1107/S2052252519007395/mf5034sup1.pdf

## Figures and Tables

**Table 1:** Size comparison of crystals grown via conventional hanging-drop experiments versus crystals grown on Roadrunner II chips using the same crystallization condition In all cases protein solutions were mixed with precipitant solutions in a 1:1 ratio. The volumes used for the drop and the reservoir were 6 and 500 µl for the hanging-drop experiments and 100 µl and 5–7 ml for the on-chip crystallization experiments, respectively. Unless otherwise stated, crystallization experiments were performed at room temperature.

Sample	Crystallization condition	Hanging-drop crystals	On-chip crystals
hAQP2 (*Homo sapiens*)	9 mg ml^−1^ protein, 0.1 *M* Tris pH 8.5, 0.1 *M* NaCl, 0.1 *M* MgCl_2_, 10 m*M* CdCl_2_, 22–25% PEG 400	Different shapes and sizes, ranging from small needles to blocky rods	Different shapes and sizes, ranging from small 10 µm needles to larger 20 × 250 µm needles and up to 50 × 150 µm blocky rods
CODH (*Oligotropha carboxidovorans*)	19.3 mg ml^−1^ protein, 0.75 *M* KH_2_PO_4_/KOH pH 7.5, 0.75 *M* NaH_2_PO_4_/NaOH pH 7.5, 94 m*M* HEPES/NaOH pH 7.5, 3% MPD at 4°C	50–100 µm wide, blocky, rod-shaped	50–100 µm wide, blocky, rod-shaped
Lysozyme (*Gallus gallus*)	80 mg ml^−1^ protein, 0.1 *M* sodium acetate pH 4.8, 12% NaCl, 20% ethylene glycol	50–170 µm, cuboid	Up to 170 µm, cuboid
Lysozyme (*Gallus gallus*)	60 mg ml^−1^ protein, 50 m*M* sodium acetate pH 3.5, 0.75 *M* NaCl, 30% ethylene glycol, 11.25% PEG 400 at 4°C	Up to 180 µm, cuboid	Up to 170 µm, cuboid
Proteinase K (*Tritirachium album*)	20 mg ml^−1^ protein, 0.1 *M* CHC buffer pH 6.5, 10 m*M* CaCl_2_, 0.7 *M* ammonium sulfate	40–50 µm, rhomboid	Up to 80–125 µm, rhomboid
Thaumatin (*Thaumatococcus daniellii*)	10 mg ml^−1^ protein, 0.1 *M* ADA pH 6.5, 0.9 *M* sodium/potassium tartrate	Up to 140 µm, rhomboid	Up to 120 µm, rhomboid
Thermolysin (*Geobacillus stearothermophilus*)	22.5 mg ml^−1^ protein, 0.1 *M* MES–NaOH pH 6.5, 10 m*M* CaCl_2_, 5% PEG 2000	Up to 60 × 300 µm, rod-shaped	Up to 60 × 320 µm, rod-shaped

**Table 2:** Diffraction and refinement data statistics for proteins crystallized and soaked on Roadrunner I chips

	Proteinase K	Thermolysin	Thermolysin–aspartate	DRAK2–ADP
PDB code	6qf1	6qf2	6qf3	6qf4
Data-collection temperature (K)	298	100	100	100
No. of crystals merged	3	3	5	4
Space group	*P*4_3_2_1_2	*P*6_1_22	*P*6_1_22	*P*4_1_22
*a*, *b*, *c* (Å)	68.47, 68.47, 108.27	92.67, 92.67, 128.31	93.15, 93.15, 129.29	85.48, 85.48, 118.07
α, β, γ (°)	90, 90, 90	90, 90, 120	90, 90, 120	90, 90, 90
Resolution range (Å)	44.20–1.74 (1.77–1.74)	46.33–1.73 (1.77–1.73)	46.58–1.52 (1.55–1.52)	42.74–2.50 (2.60–2.50)
*R*_meas_	0.150 (0.677)	0.175 (1.013)	0.121 (0.932)	0.113 (0.522)
Mean *I*/σ(*I*)	10.9 (3.4)	11.4 (3.1)	21.6 (4.7)	14.7 (5.3)
Completeness (%)	99.6 (99.9)	100 (100)	100 (99.6)	98.6 (99.0)
No. of unique reflections	27136 (1465)	34527 (1852)	51432 (2484)	15529 (1743)
Multiplicity	11.1 (11.3)	13.2 (13.6)	29.1 (29.2)	9.3 (9.7)
CC_1/2_	0.997 (0.921)	0.995 (0.810)	0.999 (0.939)	0.997 (0.992)
Mosaicity (°)	0.040	0.200	0.150	0.210
Wilson *B* factor (Å^2^)	13.37	12.63	11.90	40.22
Resolution for refinement	44.20–1.74 (1.78–1.74)	46.33–1.73 (1.79–1.73)	46.58–1.52 (1.56–1.52)	42.74–2.50 (2.58–2.50)
No. of reflections used	27060 (1765)	34457 (2936)	51351 (3451)	15521 (1256)
No. of reflections used for *R*_free_	1995 (140)	1571 (139)	1939 (135)	1552 (139)
*R*_work_/*R*_free_	0.134/0.156 (0.185/0.214)	0.128/0.161 (0.165/0.204)	0.123/0.150 (0.152/0.178)	0.183/0.227 (0.239/0.259)
R.m.s.d., bond lengths (Å)	0.005	0.006	0.010	0.002
R.m.s.d., bond angles (°)	0.746	0.841	1.162	0.476
Mean *B* factor (Å^2^)	17.61	16.57	16.06	57.04

**Table 3:** Data-collection and refinement statistics for human aquaporin 2 crystallized on Roadrunner II chips

PDB code	6qf5
Data-collection temperature (K)	298
No. of hits	3377
Indexed patterns	2903
Space group	*P*4_2_
*a*, *b*, *c* (Å)	122.2, 122.2, 94.14
α, β, γ (°)	90, 90, 90
Resolution range (Å)	37.38–3.70 (3.76–3.70)
*R*_split_	0.437 (1.538)
Mean *I*/σ(*I*)	2.32 (0.92)
Completeness (%)	99.97 (99.93)
No. of unique reflections	14866 (1462)
Multiplicity	28.1 (17.7)
CC*	0.937 (0.435)
Wilson *B* factor (Å^2^)	93.37
Resolution for refinement	33.89–3.70 (3.82–3.70)
No. of reflections used	14805 (1110)
No. of reflections used for *R*_free_	1488 (127)
*R*_work_/*R*_free_	0.282/0.336 (0.398/0.419)
R.m.s.d., bond lengths (Å)	0.004
R.m.s.d., bond angles (°)	0.761
Mean *B* factor (Å^2^)	105.45
