# Supplementary material for: On-chip crystallization for serial crystallography experiments and on-chip ligand-binding studies
Source: IUCrJ. 2019 Jun 19;6(Pt 4):714–28. doi: 10.1107/S2052252519007395 (PMC6608620; doi:10.1107/S2052252519007395)
Supplement: Supplementary file 1 [file m-06-00714-sup1.pdf]

IUCrJ

Volume 6 (2019)

Supporting information for article:

On-chip crystallization for serial crystallography experiments and on-chip ligand-binding studies

Julia Lieske, Maximilian Cerv, Stefan Kreida, Dana Komadina, Janine Fischer, Miriam Barthelmess, Pontus Fischer, Tim Pakendorf, Oleksandr Yefanov, Valerio Mariani, Thomas Seine, Breyan H. Ross, Eva Crosas, Olga Lorbeer, Anja Burkhardt, Thomas J. Lane, Sebastian Guenther, Julian Bergtholdt, Silvan Schoen, Susanna Törnroth-Horsefield, Henry N. Chapman and Alke Meents

S1. Structure of thermolysin crystallized on a Roadrunner I chip

We solved the structure of thermolysin (TLN) crystallized on a Roadrunner I chip up to 1.73Å resolution, using data measured at 100K at the PETRA III P11 beamline and PDB entry 2TLX as template for molecular replacement. Our structural model is basically identical to the available native structure of TLN (PDB ID: 2TLX, English et al., 1999) with an r.m.s.d. of 0.273Å for all main chain atoms. The largest difference is observed for Lys182 close to one of the Ca^{2+} binding sites, with its alpha carbon shifted by 1.24Å. However the adjacent residues are not significantly affected by this local conformational change.

At the active site, the Zn^{2+} ion shows the tetrahedral coordination typically observed in the apo form of TLN, including interactions with the side chains of Glu166, His142 and His146 and a hydrolytic water molecule (Suppl. figure 1 a). Additional spherical electron density is observed between Glu166, the outer-shell residues His 231 and Tyr157 and the hydrolytic water molecule (Suppl. figure 1 b). Analysis of anomalous data measured at three different wavelengths around the Zn K-edge (data not shown) following the procedure of Thorn et. al (Thorn & Sheldrick, 2011) proves this site to be a second Zn^{2+} ion binding site with lower occupancy. It was already described to be observable when TLN is crystallized in the presence of excess zinc (Holland *et al.*, 1995). As we did not purify the protein prior to crystallization and did not add any Zn^{2+} to the crystallization cocktail, there are most probably Zn^{2+} ion impurities present in the purchased protein lyophylisate. Moreover, the side chains of the catalytically important residues Glu166 and Tyr157 shows an alternate conformation in our structure, which is compatible with the inhibiting effect of excess zinc on TLN.

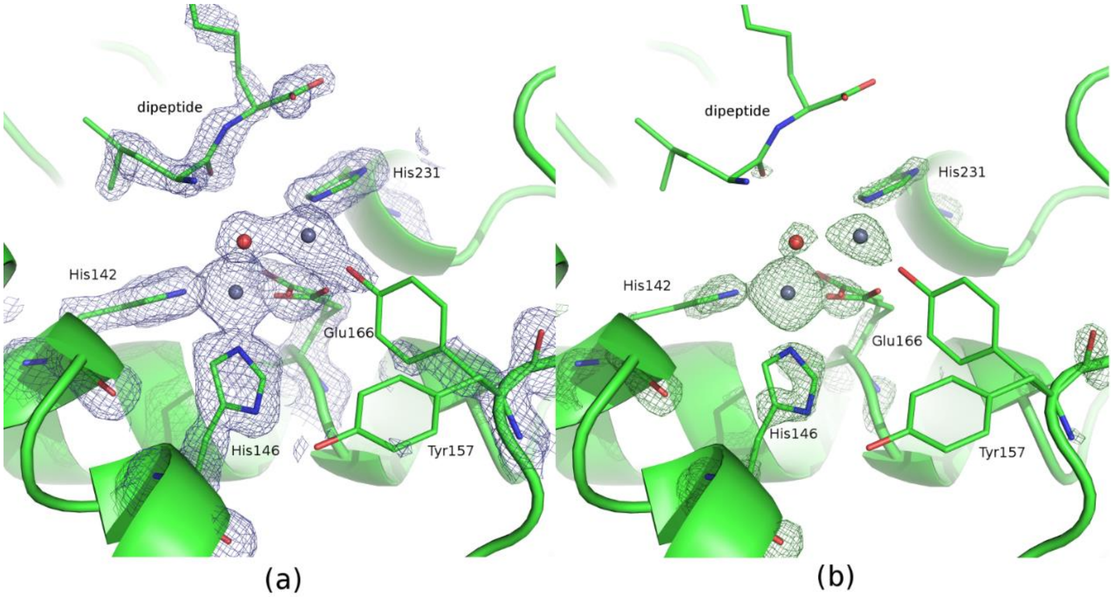

Figure S1 Active site of the TLN structure. Amino acids are shown as green sticks, two Zn^{2+} ions and the hydrolytic water molecule are represented by gray and red spheres, respectively. (a) The $2mF_o$-DF_c map at 1.5σ is shown as blue grid. The catalytically essential Zn^{2+} ion is tetrahedrally coordinated by His142, His146, Glu166 and the hydrolytic water molecule. The usually bound dipeptide, a purification artifact, is observed, but the electron density map rather supports the presence of a leucine than a valine residue at the first position. (b) The simulated annealing omit mF_o-DF_c map, shown here as green grid at 3σ, clearly indicates the presence of an additional ion binding site between His231 and the delocalized Tyr157. In accordance with anomalous data and previous conclusions of other research groups we interpret this as a less occupied second Zn^{2+} binding site.

S2. Structure of human aquaporin 2 (hAQP2) crystallized on a Roadrunner II chip

Human AQP2 is a membrane protein that forms a homotetramer in the protein crystal as well as in the biologically active structure, with each protomer fulfilling its function as a water conducting pore during the hormon-regulated water reabsorption process in the kidney collecting duct. We solved the crystal structure of hAQP2 up to a resolution of 3.7Å, using on-chip crystallization on Roadrunner II chips. Room-temperature data was collected at the MFX experimental end station at LCLS.

The comparison of our on-chip crystal structure with the 2.75Å reference structure available in the protein database (PDB ID: 4NEF, Frick et al., 2014), which was solved by conventional cryo-crystallography, reveals no major differences in the overall conformation of the

tetramer. Especially the predominantly α-helical protein cores of both structures, built by the 4x6 transmembrane helices, superimpose well (r.m.s.d. = 0.622Å for 1936 main chain atoms), whereas the surface loops show some flexibility. In general, the structures are similar with an overall r.m.s.d. value of 0.928Å for all 3596 main chain atoms. Differences are observed at the C-termini of the monomers. Whereas the C-terminal helices of three out of four monomers are resolved in the cryo-structure, in our room-temperature structure only the C-terminal helix, that is stabilized by a Cd^{2+} ion, is resolved. Whether this is a temperature effect or results from the low resolution of the data is not clear.

The mean B-factor of our room-temperature structure is with 107.9Å^2 a little bit higher compared to 91.2Å^2 for the reference cryo structure. In general, both structures exhibit a well-ordered core region, comprising the central transmembrane helices, and show higher flexibility in the loop regions, especially in the extracellular part (Suppl. figure 2 a-d). In both structures, the Cd^{2+}-stabilized loop D shows less flexibility than the equivalent loops D in the other three monomers of the tetramer, which might also be an effect of the binding of a C-terminal helix from a symmetry-related hAQP2 molecule in the groove next to loop D (Suppl. figure 2 (e-f)).

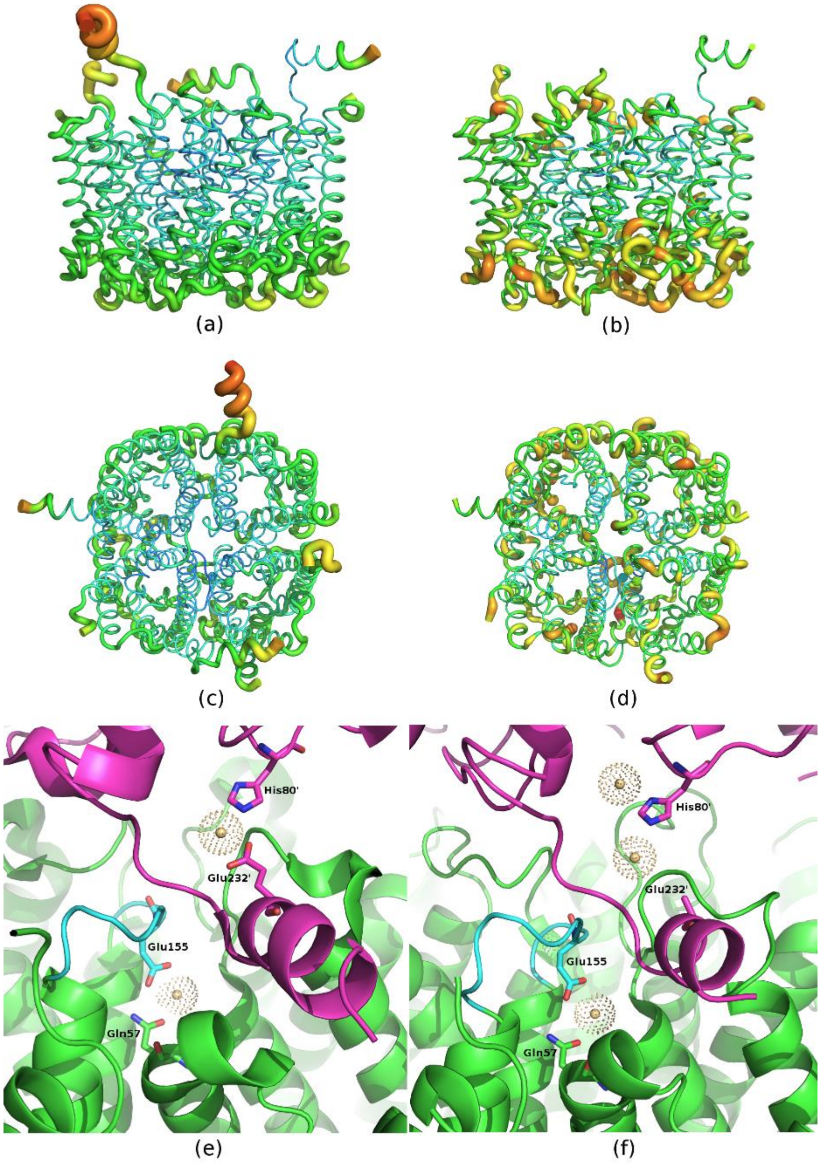

Figure S2 Structural comparison of the cryo-temperature reference structure (PDB ID: 4NEF; a, c, e) and our room-temperature structure of hAQP2 (b, d, f). (a-d) Side and top view of the tetrameric assembly in tube-representation. B-factors are represented by colors from low values in blue to high values in red as well as by tube thickness. The loop regions, especially at the extracellular side (a/b bottom), show higher flexibility than the central region comprising the 4x6 transmembrane helices. Our room-temperature structure generally exhibits higher B-factors than the cryo-temperature structure. (e-f) Close-up view of the Cd^{2+}-stabilized loop D (cyan) and the C-terminal helix of a symmetry-related hAQP2 molecule (purple) bound to the groove next to loop D. Cd^{2+} ions are shown as golden spheres and coordinating residues are shown in stick-representation. The side chain of Glu232' in our structure was not modelled due to missing electron density.

The reference crystal structure contains two Cd^{2+} ions, that are thought to substitute naturally occuring and functionally important Ca^{2+} ions. The first Cd^{2+} ion is coordinated by residues of loop D of one hAQP2 monomer. The second Cd^{2+} ion seems to stabilize the conformation of the C-terminal helix which binds to the groove next to loop D of the symmetry-related hAQP2 molecule and which was postulated to be important for hAQP2 tetramer interaction (Suppl. figure 2 e-f). Both Cd^{2+} ion binding sites are now confirmed by our structure, with the second site showing a possible alternate coordination of residue His80.

In general, the resolution of our room-temperature structure is too low to draw any conclusions from loop or side chain conformations. Further experiments have to elucidate, whether the hAQP2 structure at near-physiological temperature exhibits any significant and functionally relevant conformational differences compared to the hAQP2 structure at cryogenic temperatures.

S3. Ligand-bound structures from on-chip ligand soaking experiments

To demonstrate the applicability of the on-chip crystallization chambers for ligand soaking experiments, which takes advantage of the direct accessibility of naked crystals on silicon chips, we performed soaking experiments with on-chip crystallized TLN and in-batch crystallized DRAK2 crystals.

Apo crystals of TLN were grown directly on Roadrunner I chips and afterwards soaked with 200mM sodium aspartate dissolved in mother liquor, using our on-chip crystallization chambers in both steps. Diffraction data was measured at cryogenic temperatures. Structure solution and refinement was performed with a 1.52Å resolution dataset merged from five crystals, using the native TLN structure 2TLX as template for molecular replacement.

At the active site, the catalytic zinc ion is penta-coordinated involving residues Glu166, His 142 and His146 as well as both the amino and carboxyl group of one aspartate ligand (Suppl figure 3 a/b). Additional interactions are observed with the catalytically important outer-shell residues His 231, Glu143 and Tyr157. We interpret the density next to the aspartate side chain as a sodium ion, because we used sodium aspartate for the soaking experiment and due to the close proximity to the negatively charged carboxyl group of the aspartate (2.4Å). On the other

side, the sodium ion interacts with the main chain amide of Trp115. Comparison with the TLN structure in complex with asparagine (PDB ID: 4M65, Yin et al., 2014) reveals that both ligands bind in a very similar fashion. They just differ slightly in their side chain conformation, because asparagine interacts directly with the main chain amide of Trp115 and not mediated via a solvent molecule (Suppl. figure 3 c). In our structural model there is a second, less-occupied aspartate molecule bound directly next to the first one. It interacts with the sidechains of Asn112, Glu143 and Arg203. Inspection of the TLN-asparagine structure 4M65 reveals, that the density at this position has been interpreted as an ethylene glycol molecule. In general, the electron density maps at the second aspartate binding site are not unambiguous and the interpretation is challenging. We decided for the described binding mode on the basis of the simulated annealing composite omit map, that is thought to be most unbiased.

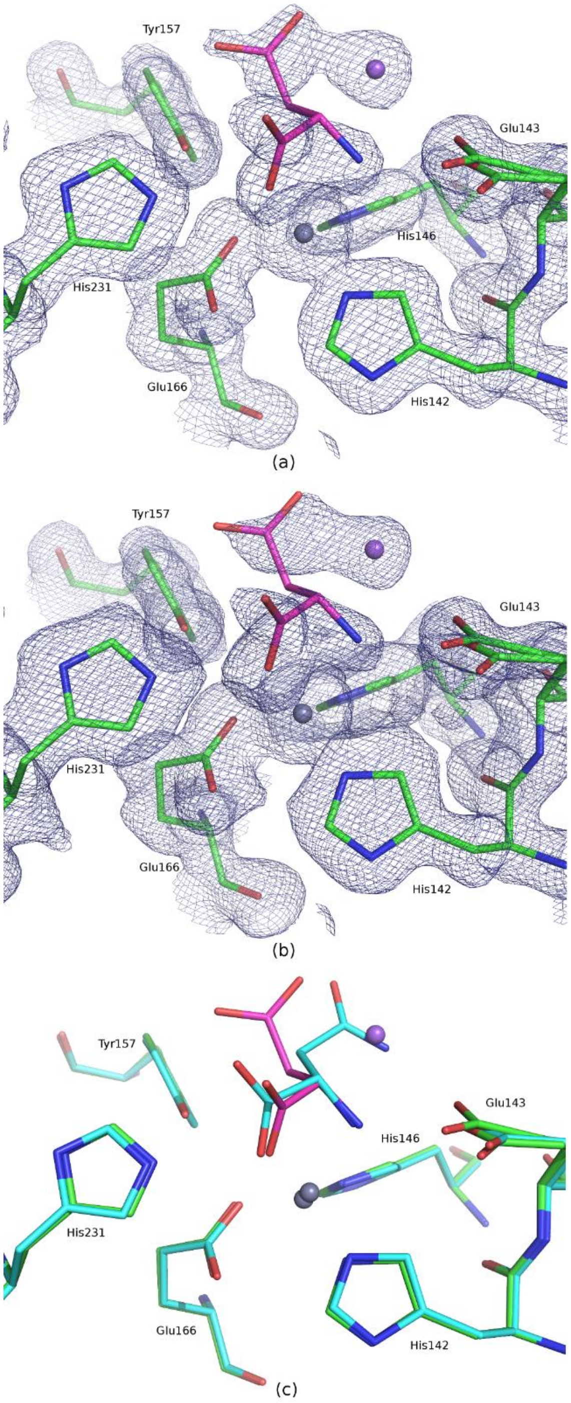

Figure S3 Active site of the TLN structure in complex with aspartate. The protein is shown in green, the ligand in purple stick representation. Gray and purple spheres represent Zn^{2+} and Na^{+} ions, respectively. (a) The $2mF_o$-DF_c electron density map at 1σ is shown as blue grid. The aspartate ligand is involved in the fivefold coordination of the Zn^{2+} ion. (b) The simulated annealing composite omit $2mF_o$-DF_c map at 1σ proves the presence of the sodium aspartate ligand at the active site. (c) Superposition of the active sites of our TLN structure in complex with sodium aspartate and the TLN structure in complex with asparagine (PDB ID: 4M65).

Both ligands interact in the same way with the catalytic Zn^{2+} ion, but differ in their side chain conformation.

In-batch grown crystals of DRAK2, a serine/threonine kinase involved in autoimmune diseases and cancer (Sanjo *et al.*, 1998; Doherty *et al.*, 2009; Yang *et al.*, 2012; Edwards *et al.*, 2015; Harris & McGargill, 2015), were soaked on the chip for one hour with its natural ligand ATP using our on-chip crystallization chamber. Cluster analysis of the collected data with BLEND led to a 2.5Å dataset merged from four individual crystals. The crystal structure was solved via molecular replacement using the quercetin-bound structure of DRAK2 available in the PDB (PDB ID: 3LM5) as search model. At the active site, the electron densitiy for the nuctleotide and one coordinated magnesium ion is clearly visible, although missing density for the gamma phosphate indicates that ATP was hydrolyzed to ADP during incubation (Suppl. figure 4).

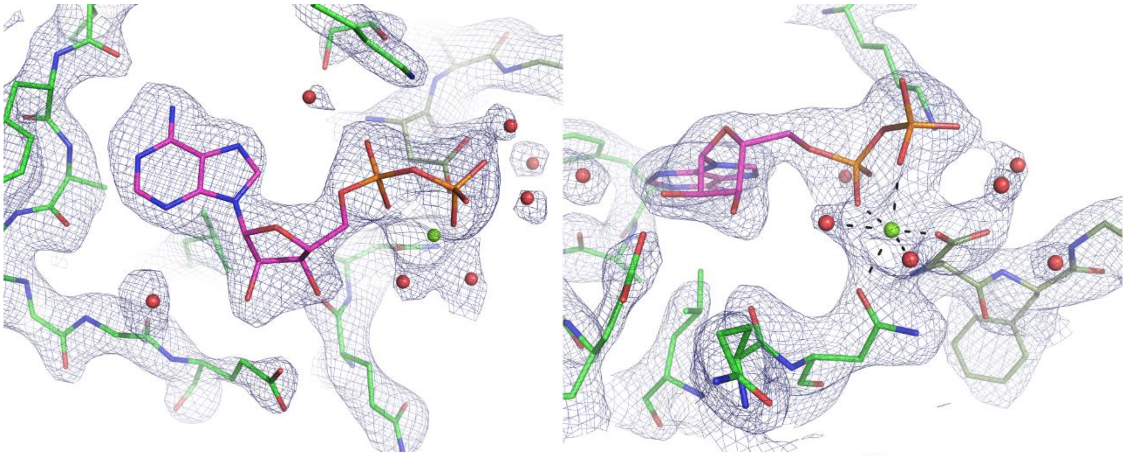

(a) (b)

Figure S4 Nucleotide binding site of the DRAK2-ADP structure. (a) The simulated annealing $2mF_o$-DF_c composite omit map (blue mesh, 1.5σ) unambiguously shows that ATP was hydrolyzed to ADP (purple) during crystal soaking, as there is no density for the γ-phosphate. (b) The magnesium ion (yellow sphere) is octahedrally coordinated by the ADP phosphates, Asp179 of the DFG-motif (dark green), the side chain of Asn163 and two water molecules.

The binding mode of the Mg-ADP complex to the active site of DRAK2 is nearly identical as observed in the ADP-complex structures of the closely related kinase DAPK1, e.g. PDB ID 2W4K or 3F5G. However, it must be noted that the active site region in our DRAK2-ADP

structure is much less compact then in the DAPK1-ADP structures (Suppl. figure 5). This is indicated for example by an elongated hydrogen bond between the Asp179 side chain and the Gly181 amide (3.6Å instead of 3.1-3.3Å), both residues belonging to the highly conserved DFG motif, or the weakened Glu80-Lys62 salt bridge (4.4Å instead of 2.8Å), which anchors the αC helix to the N-lobe in the active conformation of the kinase.

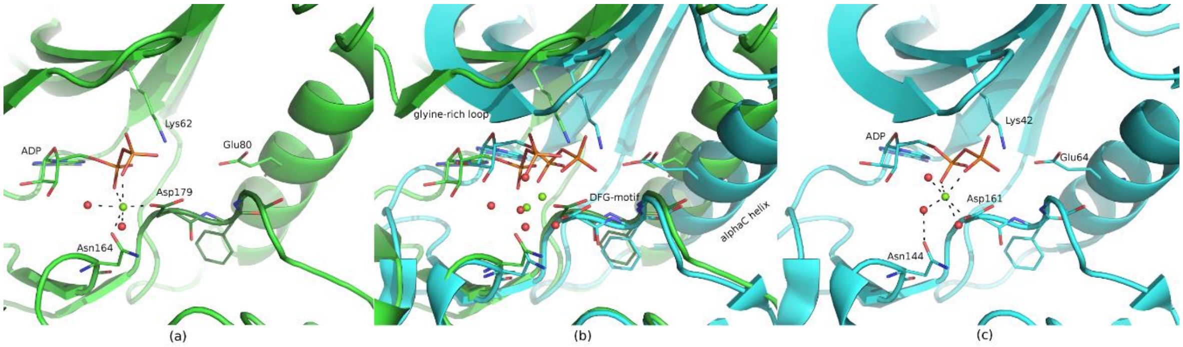

Figure S5 Comparison of the nucleotide binding sites of DRAK2 (green) and DAPK1 (cyan, PDB ID: 2W4K) in the presence of ADP. The structures were superimposed by their C-terminal domains including the conserved and catalytically important DFG-motif. Whereas in DRAK2, the magnesium ion (yellow sphere) at the active site is coordinated by the ADP phophate groups, Asp179 of the DFG -motif, two water molecules and the side chain of Asn164 (a), the interaction between the magnesium ion and the structurally equivalent residue Asn144 is water-mediated in DAPK1 (c). The active site in DAPK1 is much more compact (b), as can be seen at the position of the glycine-rich loop and the salt bridge, that is formed between the β3 Lys and the αC-helix Glu in the active conformation of the kinase (4.4Å and 2.8Å in DRAK2 and DAPK1, respectively).

The global fold of our DRAK2-ADP structure is very similar to the kinase structure inhibited by quercetin with an r.m.s.d. of 0.508Å for all main chain atoms. Even though the highly conserved DFG-motif is in the in-position, which is necessary for catalytic activity, both structures represent an inactive conformational state of the kinase with a disordered or partially disordered activation loop, a slightly displaced αC helix and a weak and hence no-longer stabilizing Lys62 (β3)-Glu80 (αC helix) salt bridge. The activation loop as well as the C-terminus of our structure show less flexibility than in the quercetin-bound structure, thus allowing for modeling five and seven additional residues, respectively.

We observe structural differences at the C-terminus between DRAK2 and DAPK1 (Suppl. figure 6), which possibly result from a different C-terminal domain organisation of these kinases. DAPK1 contains a C-terminal autoregulatory domain (ARD) with several autophosphorylation sites, that is known to be a Ca^{2+}/calmodulin binding site. As seen in the DAPK1 Mg^{2+}-ADP structure (PDB ID: 2W4K, Temmerman et al., 2014) and the DAPK1 calmodulin-bound structure (PDB ID: 2X0G, de Diego et al., 2010), the C-terminus is folding back onto the globular catalytic domain, thereby interacting with helix αD and shifting it aside. In our DRAK2 structure the C-terminus, which does not possess an ARD, projects away from the catalytic domain, stabilized by main chain interactions with symmetry-related molecules, and the helix αD together with the adjacent αDE loop show higher flexibility than the rest of the protein structure, indicated by higher B-factors and less defined electron density.

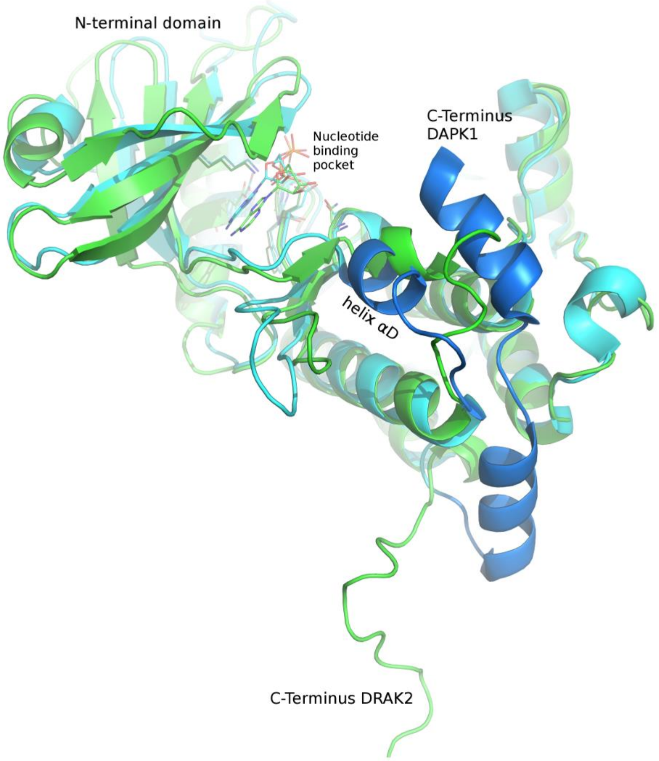

Figure S6 DRAK2 (green) and DAPK1 (PDB ID: 2W4K in cyan/blue) were superimposed by their C-terminal domain. Whereas the C-terminus of DRAK2 is unstructured and points away from the globular catalytic domain, the C-terminus of DAPK1 folds back onto it, thereby shifting helix αD and the following loop away. Helix αD, the adjacent αDE loop and the C-terminus of DAPK1 are shown in dark blue.

de Diego, I., Kuper, J., Bakalova, N., Kursula, P. & Wilmanns, M. (2010). *Sci. Signal.* **3**, ra6.

Doherty, G., Byrne, S., Austin, S., Scully, G., Sadlier, D., Neilan, T., Kay, E., Murray, F. & Fitzgerald, D. (2009). *Br. J. Cancer*. **101**, 483–491.

Edwards, B. A., Harris, T. L., Floersh, H., Lukens, J. R., Zaki, M. H., Vogel, P., Kanneganti, T. D., Bui, J. D. & McGargill, M. A. (2015). *Int. Immunol.* **27**, 161–166.

English, A. C., Done, S. H., Caves, L. S., Groom, C. R. & Hubbard, R. E. (1999). *Proteins*. **37**, 628–640.

Frick, A., Eriksson, U. K., de Mattia, F., Oberg, F., Hedfalk, K., Neutze, R., de Grip, W. J., Deen, P. M. T. & Törnroth-Horsefield, S. (2014). *Proc. Natl. Acad. Sci. U. S. A.* **111**, 1–6.

Harris, T. L. & McGargill, M. A. (2015). *PLoS One*. **10**.

Holland, D. R., Hausrath, A. C., Juers, D. & Matthews, B. W. (1995). *Protein Sci.* **4**, 1955–1965.

Sanjo, H., Kawait, T. & Akira, S. (1998). *J. Biol. Chem.* **273**, 29066–29071.

Temmerman, K., de Diego, I., Pogenberg, V., Simon, B., Jonko, W., Li, X. & Wilmanns, M. (2014). *Chem. Biol.* **21**, 264–273.

Thorn, A. & Sheldrick, G. M. (2011). *J. Appl. Crystallogr.* **44**, 1285–1287.

Yang, K.-M., Kim, W., Bae, E., Gim, J., Weist, B. M., Jung, Y., Hyun, J.-S., Hernandez, J. B., Leem, S.-H., Park, T., Jeong, J., Walsh, C. M. & Kim, S.-J. (2012). *CellReports*. **2**, 1286–1299.

Yin, X., Scalia, A., Leroy, L., Cuttitta, C. M., Polizzo, G. M., Ericson, D. L., Roessler, C. G., Campos, O., Ma, M. Y., Agarwal, R., Jackimowicz, R., Allaire, M., Orville, A. M., Sweet, R. M. & Soares, A. S. (2014). *Acta Crystallogr. D. Biol. Crystallogr.* **70**, 1177–1189.
